# Recent advances in understanding photosynthesis

**DOI:** 10.12688/f1000research.9744.1

**Published:** 2016-12-21

**Authors:** Ulf-Ingo Flügge, Peter Westhoff, Dario Leister

**Affiliations:** 1 Cologne Biocenter, Botanical Institute II and Cluster of Excellence on Plant Sciences (CEPLAS), University of Cologne, Cologne, Germany; 2 Department of Biology and Cluster of Excellence on Plant Sciences (CEPLAS), Heinrich-Heine University, Düsseldorf, Germany; 3 Plant Molecular Biology, Department of Biology I, Ludwig-Maximilians-University, Munich, Germany

**Keywords:** light-dependent reactions, assimilation reactions, photosynthetic organisms

## Abstract

Photosynthesis is central to all life on earth, providing not only oxygen but also organic compounds that are synthesized from atmospheric CO _2_ and water using light energy as the driving force. The still-increasing world population poses a serious challenge to further enhance biomass production of crop plants. Crop yield is determined by various parameters, *inter alia* by the light energy conversion efficiency of the photosynthetic machinery. Photosynthesis can be looked at from different perspectives: (i) light reactions and carbon assimilation, (ii) leaves and canopy structure, and (ii) source-sink relationships. In this review, we discuss opportunities and prospects to increase photosynthetic performance at the different layers, taking into account the recent progress made in the respective fields.

## Introduction

Photosynthesis is a process that all life on earth depends on. Photosynthetic organisms convert more than 10 ^9^ metric tons of atmospheric CO _2_ into biomass per year. With the global human population rising from ~7 billion now to 9–10 billion by 2050, the worldwide trend towards a more meat-rich human diet, the loss of harvest and grazing land, and the negative effects of global warming on crop production have put forward the question of whether this incredibly high amount of biomass production can be further increased.

Crop yield is determined by the available solar irradiation energy across the growing season (0.487 S _t_), the genetically encoded properties of how the radiation is intercepted (ε _i_), the efficiency by which the light energy is converted into biomass (ε _c_), and what fraction of the total biomass is partitioned into the harvestable part of the plant (harvest index, ε _p_). This results in the Monteith equation: yield = 0.487 • S _t_ • ε _i_• ε _c_• ε _p_^1– 3^.

The *Green Revolution* raised the yield potential of the major grain crops mainly by increasing the harvest index, which is now about 0.6. Breeders were also able to improve the light interception efficiency, which in modern cultivars is up to about 0.8–0.9. All available evidence suggests that additional grain yields by further increasing the harvest index or optimizing light interception are rather unlikely; they appear to be close to their biological limits already. In contrast, the best light conversion efficiency (ε _c_) observed in field experiments (0.24 in C _3_- and 0.37 in C _4_-crops) is far below the theoretical maxima (0.46 in C _3_- and 0.6 in C _4_-plants) and thus not yet close to its biological limit. Enhancing, and in the long term re-designing, photosynthesis with respect to light energy conversion efficiency is therefore a prime target when aiming to increase crop yield ^3– 6^.

**Figure 1.**
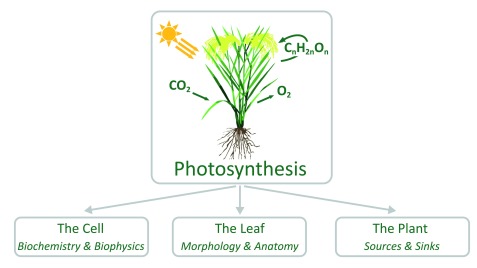
The process of photosynthesis viewed from different perspectives.

In general, photosynthesis can be described as a cellular trait that uses light energy to convert atmospheric CO _2_ into carbohydrates. At a higher level, photosynthesis is determined by the activity of leaves and by the canopy structure. And, finally, photosynthesis is related to the capacities of source and sink tissues, i.e. mature leaves and heterotrophic organs, respectively ( Figure 1).

Here, we will discuss the process of photosynthesis from these various perspectives and address putative targets for the improvement of photosynthetic performance.

## Photosynthesis as a cellular trait: (1) light-dependent reactions

### Novel insights into the dynamic interactions of photosynthetic complexes

The light-dependent reactions involve five major multi-protein complexes: photosystem I (PSI), photosystem II (PSII), cytochrome b _6_f, ATP synthase, and NADPH dehydrogenase (NDH). In particular, PSI can (transiently) form larger super-complexes with other complexes like cytochrome b _6_f ^7^, NDH ^8^, and PROTON GRADIENT 5 (PGR5)-PGR5-LIKE 1 (PGRL1) ^9, 10^. More recently, interactions of PSI with PSII ^11^ and light-harvesting complex II (LHCII) ^12^ have been characterized. Traditionally, PSI and PSII are thought to be spatially dispersed. However, in cyanobacteria, a megacomplex consisting of PSII, PSI, and phycobilisomes was found ^13^. Aro and co-workers reported that PSI and PSII complexes coexist with LHCs in *Arabidopsis* ^14^. More recently, Tanaka and colleagues suggested that about half of PSIIs are physically connected to PSI complexes ^11^ *.* The obtained results suggest that when PSII becomes excessively excited, it can divert excitation energy directly to PSI to avoid photo-damage. Conversely, photoinhibition of PSI can downregulate PSII via a mechanism that involves increased reduction of the intersystem electron carrier system due to damage to the FeS clusters of PSI. This is associated with the activation of thylakoid phosphorylation-based mechanisms that increase energy flow towards PSI ^15^.

Bassi and colleagues have recently generated plants with a PSI that lacks its natural LHCI antenna system ^12^. Despite the absence of LHCI, LHCII can still attach to PSI, and in fact each LHCI-less PSI complex binds one LHCII trimer, thus fully replacing the four Lhca proteins from LHCI by the three Lhcb proteins of LHCII. This demonstrates that LHCI is not necessary for excitation energy transfer from LHCII to PSI, as was previously suggested ^16^. Intriguingly, the transfer of energy from LHCII to PSI appears to be more efficient than from LHCI, raising the question of why PSI uses LHCI instead of LHCII. A plausible explanation is that LHCI absorbs photons with lower energy than LHCII, avoiding competition between PSI and PSII for the same photons. Therefore, in low-intensity and far-red enriched light (like the one available under canopy cover), PSI is favored over PSII, avoiding over-reduction of plastoquinone and thus preventing photo-damage.

With respect to photosynthetic improvements, the structural flexibility of the photosynthetic complexes might allow the construction of novel combinations of photosystem cores and LHC antenna complexes. Indeed, it appears to be possible to design an entire set of such combinations that could be tested under different light regimes for their efficiency and use under controlled (such as in greenhouses) and natural (such as in the field) light conditions. In a complementary approach, conditional regulation of various light-harvesting genes might allow for tuned optimization in an ever-changing and challenging environment. Therefore, biotic interactions also need to be considered, taking into account, for instance, trade-offs between photosynthesis and herbivore resistance ^17^.

### Novel auxiliary components of photosynthetic light reactions

During the last few decades, the structural components of the light reactions, including the multi-protein complexes PSI and PSII, cytochrome b _6_f, ATP synthase, and NDH, have been identified and extensively characterized at the gene, protein, and mutant level. In contrast, the inventory of the auxiliary components of the photosynthetic light reactions is far from being complete. Novel proteins involved in the biogenesis, repair, regulation, and protection of the photosynthetic machinery are continuously identified owing to genetic screens and functional genomics approaches and employing mainly the model species *Arabidopsis thaliana*, *Chlamydomonas reinhardtii*, and *Synechocystis* sp. PCC 6803. In Table 1, recently identified auxiliary proteins of the photosynthetic light reactions are listed. Adding their numbers to the ones of the factors already identified before (reviewed in 18), it becomes clear that the number of different proteins controlling the biogenesis and repair of the multi-protein complexes exceeds the one of structural subunits. While for PSI and NDH, and particularly PSII, substantial sets of assembly factors have been known for many years already, until recently only one auxiliary protein (ALB4 ^19^) involved in the biogenesis of the chloroplast ATP synthase (cpATPase) has been isolated. Three additional cpATPase assembly factors have now been identified: CGL160 ^20, 21^, PAB ^22^, and CGLD11/BFA3 ^23, 24^. CGL160 represents a protein of prokaryotic origin, whereas CGLD11/BFA3 and PAB are specific to photosynthetic eukaryotes. Interestingly, CGLD11/BFA3 is also targeted to mitochondria but not (yet) essential for the assembly of the mitochondrial ATP synthase ^23^. Similar pronounced evolutionary dynamics are displayed by PAM68L, which evolved from a PSII assembly factor to mediate the assembly of the chloroplast NDH complex in *Arabidopsis* ^25^.

**Table 1.** Overview of novel auxiliary components of photosynthetic light reactions identified in the last few years.

Function	Protein/Species	Reference
*Biogenesis and repair*		
PSII biogenesis	AtCtpA ( *Arabidopsis thaliana*)	74
	TRX-m1, -m2 and -m4 ( *A. thaliana*)	75
	RBD1 ( *Chlamydomonasreinhardtii* , *Synechocystis*, *A. thaliana*)	76
	THF1 ( *A. thaliana*)	77, 78
	PsbN (Tobacco)	79
	AtTerC ( *A. thaliana*)	80
	CyanoP ( *Synechocystis*)	81
PSII repair	Psb28 ( *Synechocystis*)	82, 83
	Slr0151 ( *Synechocystis*)	84, 85
	MET1/TEF30 ( *A. thaliana*, *C. reinhardtii)*	86, 87
PSI biogenesis	PSA2 ( *A. thaliana*)	88
	FtsH2 and FtsH5 ( *A. thaliana*)	89
Chloroplast ATP synthase assembly	CGL160 ( *A. thaliana*)	20, 21
	PAB ( *A. thaliana*)	22
	CGLD11/BFA3 ( *A. thaliana*)	23, 24
Assembly of NADPH dehydrogenase complex	PAM68L ( *A. thaliana*)	25
	NdhP ( *Thermosynechococcus elongatus*)	90
	CRR9 ( *A. thaliana*)	91
*Regulation*		
Reversible thylakoid phosphorylation	PBCP ( *A. thaliana*)	92
Thylakoid ultrastructure	CURT1 ( *A. thaliana*)	31
Photoacclimation, cyclic electron flow, and retrograde signaling	CAS ( *A. thaliana*, *C. reinhardtii*)	33– 35
Light-harvesting pigments	HPE ( *A. thaliana*)	36
*Protection*		
PSII protection	HHL1 ( *A. thaliana*)	93
	MPH1 ( *A. thaliana*)	94
PSI protection	FKBP16-1 ( *A. thaliana*)	95

The network of protein kinases and phosphatases involved in reversible thylakoid phosphorylation (reviewed in 26) and the two ferredoxin-plastoquinone reductase complexes involved in cyclic electron flow (reviewed in 27) are now relatively well characterized, and also several factors regulating the dynamics of thylakoid ultrastructure and the acclimation and protection of the photosynthetic machinery have been identified (reviewed in 28– 30). Three recent examples are highlighted here: (i) the levels and phosphorylation states of CURT1 proteins were shown to control the formation of the appressed regions of thylakoids in the so-called grana stacks ^31^; it remains to be elucidated how the activity of CURT1 is regulated under physiological conditions. (ii) The calcium sensor CAS was localized to thylakoids ^32^, appears to regulate acclimation and cyclic electron flow in *C. reinhardtii* ^33, 34^, and was more recently associated with retrograde signaling in *A. thaliana* ^35^; here, future research needs to clarify the role of chloroplasts in cellular calcium signaling and how CAS is involved in this process. (iii) Very recently, the chloroplast-splicing factor HPE1 was shown to be involved in the regulation of photosynthetic efficiency; in fact, plants without HPE1 re-adjust their light-harvesting pigments, thereby reducing antenna size, improving light capture, decreasing energy loss, mitigating photodamage, and enhancing photosynthetic quantum yield during photosynthesis ^36^.

As suggested before, the most promising targets for genetic engineering of the light reactions of photosynthesis––in terms of manipulating one or a few genes––are modifying light harvesting and regulators of photosynthetic electron flow ^37^. The resulting plants have the potential to exhibit more efficient photosynthesis under controlled conditions, e.g. in greenhouses, or in regions that cannot otherwise be extensively used for agriculture because of their short growing seasons. In fact, plants without the LHCII protein phosphatase TAP38 and concomitantly with hyper-phosphorylated thylakoid proteins ^38^ or without HPE1 ^36^ (see above) appear to be superior to normal plants under certain conditions, implying that the modification of single regulatory proteins might positively impact photosynthesis. Moreover, since evolutionary-based adaptation of plants might not cope with the relatively rapid progression of human-made climate change that we experience today, transgenic plants with altered acclimation capacity due to altered activity of accessory photosynthetic proteins might contribute to the generation of improved crop varieties. Proof of this concept was provided very recently. The parallel overexpression of three proteins involved in photoprotection (subunit S of PSII [PsbS], violaxanthin de-epoxidase [VDE], and zeaxanthin epoxidase [ZEP]) accelerated the photoprotective response to natural shading events in tobacco, resulting in increased plant dry matter productivity in fluctuating light and under field conditions ^39^.

## Photosynthesis as a cellular trait: (2) assimilation reactions

During photosynthesis, plants capture energy from sunlight and turn it into biochemical energy in the form of ATP and reducing equivalents, NADPH. In C _3_-plants, the first product of the CO _2_-fixing ribulose-1,5-bisphosphate carboxylase/oxygenase (RuBisCO), the C _3_-compound 3-phosphoglycerate, is transformed to a C _3_-sugar (triose phosphate) as part of the Calvin-Benson cycle using both ATP and NADPH as products of the light reaction. In most plants, triose phosphates are subsequently converted into sucrose as the main photoassimilate exported from photosynthetically active leaves (the source tissue). The long-distance transport of photosynthates to heterotrophic organs (the sink tissue) serves to supply these organs with carbon and energy. In harvestable organs, carbon can be stored as, e.g., sucrose, starch, oil, or, in combination with nitrogen, protein.

In C _3_-plants, the category to which most of our crop plants belong to, RuBisCO is CO _2_ limited and works, at best, at half of its maximal reaction velocity ^40^. The oxygenase activity of RuBisCO initiates the wasteful process of photorespiration that finally results in the release of previously photoassimilated CO _2_ and ammonia ^41^. Multiple efforts have been or are still being undertaken to improve RuBisCO activity either directly ^42^, albeit with very limited success so far ^6^, or by targeting auxiliary proteins, for instance RuBisCO activase ^43^. Alternatively, by introducing CO _2_ pumping devices into C _3_-species, the limitations of RuBisCO are expected to be reduced. These CO _2_ pumps are to operate via either a C _4_ cycle ^44^ or inorganic carbon-concentrating devices of cyanobacterial origin ^45^. There were also attempts to reduce photorespiratory losses by introducing alternative salvage pathways ^46, 47^. In addition, promising approaches have also been initiated to overcome bottlenecks in the Calvin-Benson cycle ^48, 49^.

## Photosynthesis: leaves as target for photosynthesis improvement

Leaves are the plant’s organs that are dedicated to photosynthesis. Under high-light conditions, e.g. at midday, plants receive excess energy that they are not able to cope with. It was proposed earlier that photosynthetic efficiency could be maximized by improving the plants’ canopy light distribution in a way that minimizes light saturation of the upper leaves and light starvation of the lower leaves ^6^. This could be achieved by (i) varying the angle of the leaves in the canopy, (ii) altering the size of LHCs per photosystem, i.e. fewer LHCs in the upper leaves and larger LHCs in lower leaves, and/or (iii) extending the light absorption spectrum of photosynthetic pigments into the near-infrared region in the lower leaves in order to use this light quality more efficiently ^6, 50, 51^.

The leaves of the angiosperms vary greatly in size and shape, from single and entire to highly complex compound leaves ^52^. However, the role and importance of leaf anatomy in contributing to the photosynthetic output of leaves is largely unexplored. It was suggested earlier that both leaf shape and leaf anatomy contribute to the photosynthetic output of leaves ^53^ and are therefore promising targets for improving photosynthesis ^54^. Genetic studies with rice support this notion by demonstrating that in rice the anatomy of leaves is closely associated with leaf photosynthesis ^55, 56^ and, moreover, with crop yield ^57, 58^. Unfortunately, global genetic analyses, preferably with easily accessible genetic model plants, which aim to identify genes affecting the inner anatomy of leaves and concomitantly their photosynthetic output, are still missing.

One approach to tackle this issue would be to compare leaf form and anatomy in established model systems, e.g. *A. thaliana* and *Cardamine hirsuta*, which both belong to the Brassicaceae. In contrast to *A. thaliana*, *C. hirsuta* has complex leaves subdivided into leaflets. Major steps in understanding the genetic basis for variation in leaf shape both within and between species have already been made ^59– 63^. Both species have been documented to be suitable for an easy identification of genes and their functions. In both species, genes could be identified that affect primarily leaf form and/or the structure and organization of palisade and spongy parenchyma tissues of the leaf. A second step would be to investigate how and to which degree these genes can be used to optimize the photosynthetic output of leaves. The forward genetic approaches could rely on either mutagenesis or the available natural genetic diversity. Both photosynthesis and leaf differentiation are rather conserved in evolution, at least among the angiosperms. The transfer of knowledge from these model systems to the major crop species of this plant family, namely the Brassicas, should therefore be relatively straightforward.

## Photosynthesis and the interaction with source-sink metabolism

Photosynthesis is part of a superordinate system, namely the whole plant. From the system’s perspective, the production of goods and their utilization have to be coupled, i.e. previous to the storage of products derived from photosynthetic assimilation processes in sink tissues (i.e. in harvestable organs), and carbon and nitrogen have to be assimilated in the source tissue (i.e. in mature leaves). To increase biomass production, strengthening the capacities of both source and sink tissues have been aimed at, although with varying success ^64– 71^. Molecular targets were mainly related to genes involved in sugar and starch metabolism. A promising approach was recently suggested comprising the simultaneous boosting of both source and sink capacities using the crop plant potato as an example ^72^. Here, the moderate repression of the leaf ADP-glucose pyrophosphorylase, a key enzyme of transitory starch formation or, alternatively, the mesophyll-specific expression of a bacterial pyrophosphate to stimulate sucrose synthesis and to prevent sucrose breakdown were combined with plants that had an increased sink strength. An increase in sink strength was accomplished by tuber-specific overexpression of two plastidic transporters responsible for the import of carbon (glucose 6-phosphate ^73^) and energy (ATP ^74^), respectively, into starch-storing amyloplasts. Both procedures resulted in an enhanced allocation of sucrose from source to sink tissues at the expense of leaf starch accumulation. The combined push-pull approach resulted in doubling the tuber starch yield ^72^. This way of proceeding is currently being transferred to cassava, an important staple food in developing countries (Bill & Melinda Gates Foundation, CASS, OPP1113365).

It remains to be elucidated whether or not the push-pull approach can be combined with recent knowledge on novel photosynthetic components, e.g. by novel combinations of photosystem cores and LHC antenna complexes or by modifying light harvesting and regulators of photosynthetic electron flow to improve photosynthetic efficiency ( Table 1). In addition, the push-pull strategy could potentially be teamed up with approaches to alter leaf form and/or anatomy to further improve ε _c_, i.e. photosynthetic performance and biomass production.
